# Crop Load Influences Growth and Hormone Changes in the Roots of “Red Fuji” Apple

**DOI:** 10.3389/fpls.2020.00665

**Published:** 2020-05-26

**Authors:** Bowen Liang, Yu Sun, Zhongyong Li, Xueying Zhang, Baoying Yin, Shasha Zhou, Jizhong Xu

**Affiliations:** College of Horticulture, Hebei Agricultural University, Baoding, China

**Keywords:** apple, root system, endogenous hormones, minirhizotron, fruit load

## Abstract

Crop load has a substantial impact on growth of the aerial and belowground parts of apple trees. Here, we examined the effects of different crop loads on growth and hormone levels in apple roots. A crop load of 1.5 (T1.5) fruits per cm^2^ trunk cross-sectional area (TCSA) treatment resulted in lower root growth vigor, while non-fruiting (T0) and T0.4 conditions showed higher root growth vigor. In all treatments, dead roots increased in length 90 days after full bloom (DAFB), whereas live roots were more abundant at about 50 and 170 DAFB, showing a bimodal curve. During each root growth peak, levels of cytokinins (CTKs), indole acetic acid (IAA), and gibberellic acid (GA_3_) were higher. Moreover, hormone levels gradually decreased with increasing crop load within each peak. Root turnover tended to decrease with decreasing crop load. These findings indicate that root growth and hormone contents were positively correlated during the fruit growth phase, and that the negative impact of crop load on root growth may have been caused by hormone level decreases.

## Introduction

Alternate bearing is one of the greatest problems in apple production, resulting in unbalanced production and fruit quality. This issue can be alleviated by managing an appropriate crop load, which influences vegetative and productive growth (Smith and Samach, 2013). For instance, excessive crop load may reduce shoot elongation, leaf growth, and root development, which directly affect photosynthesis and carbon allocation. Root growth is of great significance for vegetative growth, because nutrients are necessary for the energy required for both photosynthesis and root uptake. Studies investigating the effects of fruits on photosynthesis, partitioning of assimilates, and dry matter accumulation have shown higher leaf photosynthetic efficiency in fruiting than in non-fruiting trees (Heim et al., 1979; Negi and Sharma, 2011). Both roots and fruits, non- or low-photosynthetic organs, act as sinks depending exclusively on photosynthetic products imported from leaves. Thus, root growth is much greater in non-fruiting trees than in fruiting trees (McClure and Cline, 2015). Unequal competition among sinks may cause a disequilibrium between vegetative and reproductive growth due to changes in carbon allocation, which may lead to a new source/sink balance and water and nutrient supplies. Carbon distribution plays an important role in root development. Despite studies on fruit and shoot growth, the effects of crop load on root growth and development remain unclear.

Phytohormones are endogenous substances that play vital roles in plant growth and development. Hormone signals transmit information about environmental and endogenous changes to integrate the physiological responses of the whole plant to optimize growth and development, and form new source–sink balances (Lemoine et al., 2013). The initiation and development of roots, which supply water and nutrients, are affected by the combined actions of endogenous phytohormones. Plant growth is modulated by sink strength, and cytokinins (CTKs) regulate the rate-limiting steps that determine nutrient availability by establishing local metabolic sinks (Werner et al., 2008). CTKs have an inhibitory effect on lateral root initiation and a stimulatory effect on lateral root elongation. Exogenously applied CTKs completely inhibit lateral root primordia formation and stimulate lateral root elongation by increasing cell length (Goodwin and Morris, 1979). Exogenously applied auxin counteracts the effect of CTKs on lateral root initiation and elongation, suggesting that CTKs act on lateral root elongation through an auxin-dependent pathway (Debi et al., 2003). Numerous studies have shown that auxin is necessary for lateral root initiation and subsequent growth (Blakely et al., 1988; Celenza et al., 1995; Reed et al., 1998). Exogenous application of auxin or enhancement of endogenous auxin synthesis results in a significant increase in the number of lateral roots (Kares et al., 1990; Boerjan et al., 1995; Laskowski et al., 1995). CTKs, together with auxin, play an essential role in plant morphogenesis, and have a strong influence on root and shoot formation and relative growth. CTKs act antagonistically to auxin and determine cell senescence by promoting shoot and root differentiation in callus culture (Laplaze et al., 2007).

Many studies have reported on the physiological functions of gibberellins (GAs) during root growth. Concentration-dependent stimulation of elongation growth by GA is important for regulating plant height and root length. In GA depletion experiments, either by inhibiting GA biosynthesis or using GA-deficient mutants, remarkable thickening of roots was observed, while slender roots were induced by GA treatment (Tanimoto, 1987, 1994). Furthermore, GA clearly stimulated root elongation under growth-suppressed conditions induced by a GA biosynthesis inhibitor (Zhang and Hasenstein, 1999). Finally, Tanimoto (2012) showed that endogenous GA content affected the root-to-shoot ratio.

The effects of hormones on root development have been well documented (Casimiro et al., 2003; Peret et al., 2009; Petricka et al., 2012). However, few such studies have applied minirhizotron methods. Despite the large body of experimental work on exogenous hormone application to roots, little is known about the effects of crop load on endogenous hormone contents, root growth, and their interactions. Therefore, to gain a better understanding of the effects of crop load on the belowground response, we conducted an experiment using minirhizotrons, which allowed for clear, accurate, and continuous observations of root growth. Plants were grown in pots for accurate observations, and the root growth dynamics and root hormone contents under different crop loads were determined. The minirhizotron observation method allowed us to compare root length, surface area, and volume under different crop load conditions during the observation period, revealing the changes in root growth dynamics and the relationship between root growth and root hormone contents. Our hypothesis was that crop load would have a negative effect on root CTK, GA_3_, and IAA contents, thereby reducing root growth. To test this hypothesis, we assessed root growth dynamics and root hormone contents.

## Materials and Methods

### Plant Materials

The trials were conducted at Hebei Agricultural University, Baoding, Hebei, China. We used 4-year-old Tianhong 2/SH40/Baleng crabapple potted plants. All plants were grown in cylindrical root limiter (30 cm × 30 cm) filled with loam in a greenhouse under natural temperature and light conditions.

### Experimental Design

On April 19, 2018, plants of similar size (height, ∼1.5 m, trunk girth, 21 cm) were transplanted into cylindrical non-woven fabric pots (75 cm × 60 cm) filled with loam. The spacing between plants and rows was 1 m × 1.5 m. For root growth observations, two minirhizotrons (length, 60 cm; diameter, 7 cm) were installed 25 cm from the trunk on the east and west sides of each plant during transplanting. The experimental layout was completely randomized and we selected 24 plants and divided these into four crop load treatments. The plant material was thinned on May 18, and crop loads of 0 (T0), 0.4 (T0.4), 1.1 (T1.1), and 1.5 (T1.5) fruits per cm^2^ trunk cross-sectional area (TCSA) were set up. Each treatment was repeated six times. Drip irrigation was used with two drippers per pot evenly distributed on both sides of the plant. Beginning at 50 days after full bloom (DAFB) on June 8, the roots were sampled with a soil sampler every 20 days to determine hormone contents. After sampling, the samples were transported to the laboratory and cleaned in distilled water, and then put into liquid nitrogen and stored at −80°C.

### Root Data Acquisition and Analysis

The root Scanner-R root detection system was used to scan and collect root images every 20 days beginning 50 DAFB. Root analysis software was used to process the images, and the occurrence and death of new roots were observed and recorded. Relevant indicators were calculated based on unit soil volume (S × D), where S is the area of the cultivation matrix observed by a single minirhizotron (*S* = 7π × 22 cm^2^) and D is the observed thickness of the substrate (*D* = 0.25 cm). Roots that were un-suberized and white or changing to brownish in subsequent viewings were recorded as living. Roots were defined as dead when they turned black and produced no new roots on subsequent occasions (Wei et al., 2019).

Root length density (mm⋅cm^–3^) = L/(S × D), where L is the total length of a single minirhizotron. Root surface area density (mm^2^⋅cm^–3^) = SA/(S × D), and total root surface area (mm^2^) of a single micro root canal. Root volume density (mm^3^⋅m^–3^) = V/(S × D), where V is the total volume of a single micro root canal (mm^3^). Root number density (×10 m^–3^) = TN/(S × D), where TN is the total number of single micro root canals (×10^3^). The annual mortality of fine roots was calculated by the total length of dead roots per unit volume of soil, and the number of live roots was calculated by the total length of living roots per unit volume each time we observed the roots. Root turnover was estimated by the ratio of total dead root length to average live root length for the entire observation period based on the method used by Majdi and Andersson (2005).

### Determination of Hormone Content in Roots

The samples for hormones were collected under different crop loads at 50, 70, 90, 110, 130, 150, and 170 DAFB. Endogenous hormones, including indole acetic acid (IAA), zeatin riboside (ZR), dihydrozeatin riboside (DHZR), kinetin (KT), isopentenyladenine (IP), and gibberellic acid (GA_3_) contents were extracted from root samples, using a high-performance liquid chromatography method described by Fang et al. (1998). Absorbance in each well was measured at 260 nm using a microplate reader (Infinite M200; Tecan, Vienna, Austria). The extracted phytohormones were separated by nano-flow reversed-phase liquid chromatography on a nano-LC system (1260 series; Agilent Technologies, Palo Alto, CA, United States) using a nano Acquity Eclipse Plus C18 column (0.5 μm, 250 × 4.6 mm; Agilent Technologies) at a flow rate of 0.7 mL⋅min^–1^ at 30°C in a mobile phase of 40% acetic acid in 0.7% or 5% acetonitrile and 55% methanol. The samples were measured at 254 nm on a VWD Chemstation (Agilent 1260 VWD), and the retention time was 10 min. The hormones were quantified based on standard curves and expressed as ng g^–1^ fresh weight. The standard curves for each hormone were as follows: ZR (*y* = 0.0216x − 0.6876, *R*^2^ = 0.9994), DHZR (*y* = 0.0253x − 0.419, *R*^2^ = 0.9998), IP (*y* = 0.0225x − 0.0986, *R*^2^ = 0.9998), KT (*y* = 0.0213x − 0.0987, *R*^2^ = 0.9996), IAA (*y* = 0.0764x − 0.1301, *R*^2^ = 0.9998), GA_3_ (*y* = 0.4474x − 0.7624, *R*^2^ = 0.9937).

### Statistical Analysis

Experimental data are presented as the mean ± standard deviation (SD). All data were analyzed by one-way analysis of variance followed by Tukey’s multiple range test to detect differences among the groups. All statistical analyses were performed using SPSS 20.0 software (IBM Corp., Armonk, NY, United States). A *p*-value < 0.05 was considered significant.

## Results

### Effects of Different Crop Loads on Live Root Development

**FIGURE 1:**
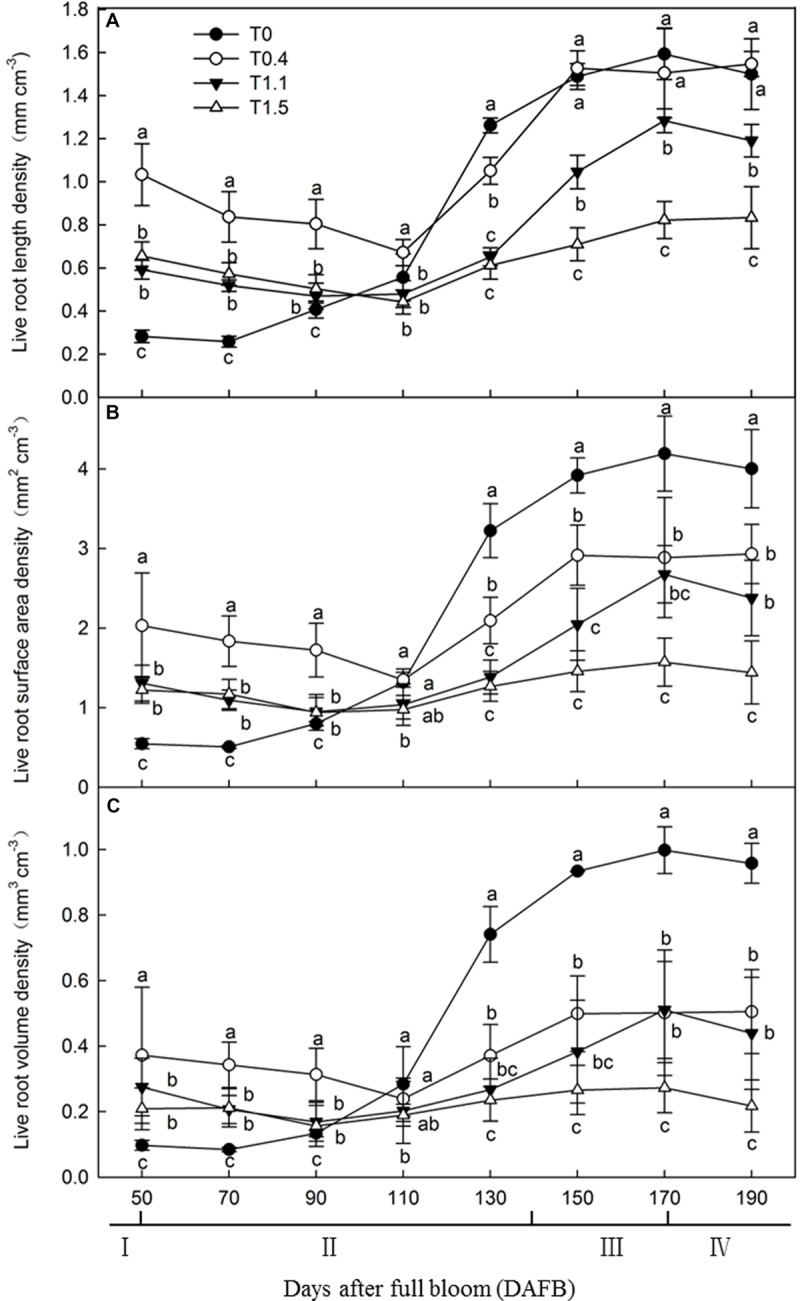
Changes in the live roots length density [LRLD **(A)**], the live root surface area [LRSAD **(B)**] and live roots volume density [LRVD **(C)**] of “Red Fuji” apple under different crop load treatments from 50 days after full bloom (DAFB) to 190 DAFB. Data are means ± SD of three replicate samples. Treatments: T0, the crop load levels of 0 fruits cm^–2^ TCSA; T0.4, the crop load levels of 0.4 fruits cm^–2^ TCSA; T1.1, the crop load levels of 1.1 fruits cm^–2^ TCSA; T1.5, the crop load levels of 1.5 fruits cm^–2^ TCSA. I, fruit set stage; II, fruit growth stage; III, fruit ripening stage; IV, fruit harvest stage. Different letters indicate significant differences between treatments, according to one-way ANOVA followed by Tukey’s multiple range test at *P*_0.05_ level.

To determine the effects of crop load on root development, we observed the roots every 20 days from 50 to 190 DAFB. Live root length density, live root surface area density (LRSAD), and live root volume density (LRVD) were determined (Figure 1). The results showed that root growth peaked at 150 DAFB under all crop loads. Among the peaks, root growth at the higher crop loads was significantly inhibited compared to that at lower crop loads. The growth peaks were less obvious after observing that the number of roots decreased from 50 to 110 DAFB. During this period, more roots were maintained under T0.4 than the other treatments. The root developing conditions of T0 differed markedly from all other treatments, showing a constantly increasing trend (Figure 1A). Root surface area and volume densities changed in a similar manner in all treatments, remaining relatively low from 50 to 110 DAFB, then increasing, and peaking at 170 DAFB. Among the peaks, LRSAD and LRVD at 130 DAFB were significantly higher in T0 and T1.5 showed the lowest levels (*p* < 0.05). The tendencies of the two parameters for each treatment were similar, although LRVD was higher than LRSAD for T0 and T0.4 (Figures 1B,C). Overall, T0 and T0.4 had longer roots than all other treatments, indicating the inhibition of root growth at higher crop loads.

### Effects of Different Crop Loads on Dead Root Length Density and Root Turnover

**FIGURE 2:**
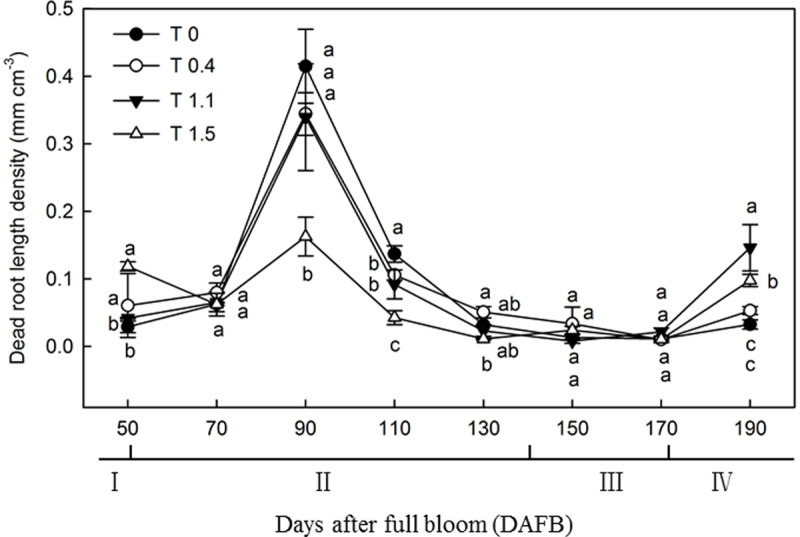
Changes in the dead root length density (DRLD) of “Red Fuji” apple under different crop load treatments from 50 to 190 DAFB. Data are means ± SD of three replicate samples. Treatments: T0, the crop load levels of 0 fruits cm^–2^ TCSA; T0.4, the crop load levels of 0.4 fruits cm^–2^ TCSA; T1.1, the crop load levels of 1.1 fruits cm^–2^ TCSA; T1.5, the crop load levels of 1.5 fruits cm^–2^ TCSA. I, fruit set stage; II, fruit growth stage; III, fruit ripening stage; IV, fruit harvest stage. Different letters indicate significant differences between treatments, according to one-way ANOVA followed by Tukey’s multiple range test at *P*_0.05_ level.

**FIGURE 3:**
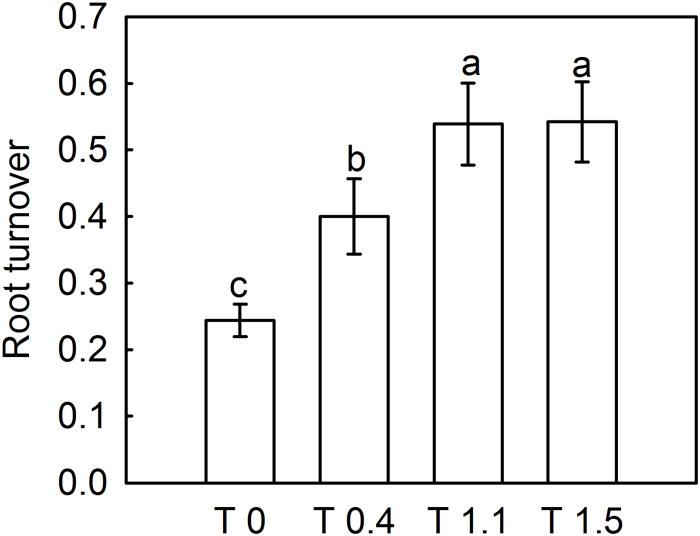
Effect of different crop load treatments on the root turnover of “Red Fuji” apple. Data are means ± SD of six replicate samples. Different letters denote significant differences at *P_0_._05_* by Tukey’s multiple range tests. Treatments: T0, the crop load levels of 0 fruits cm^–2^ TCSA; T0.4, the crop load levels of 0.4 fruits cm^–2^ TCSA; T1.1, the crop load levels of 1.1 fruits cm^–2^ TCSA; T1.5, the crop load levels of 1.5 fruits cm^–2^ TCSA.

The dead root length density (DRLD) in all treatments peaked at 90 DAFB (Figure 2), and then decreased gradually, but increased again 190 DAFB. At their peaks, T0, T0.4, and T1.1 had significantly higher DRLDs than T1.5 (*p* < 0.05). The DRLDs of T1.1 and T1.5 were significantly higher at 190 DAFB than those in T0 and T0.4, and the DRLD of T0 was significantly lower than those in the other treatments. Significant differences were observed in root turnover under different crop loads during the observation period (Figure 3). When compared with T0, the root turnover rate were increased by 66.67, 125.00, and 125.00% in T0.4, T1.1, and T1.5, respectively. Overall, root turnover presented an increasing tendency with increasing crop load.

### Effects of Different Crop Loads on Root CTK Content

**FIGURE 4:**
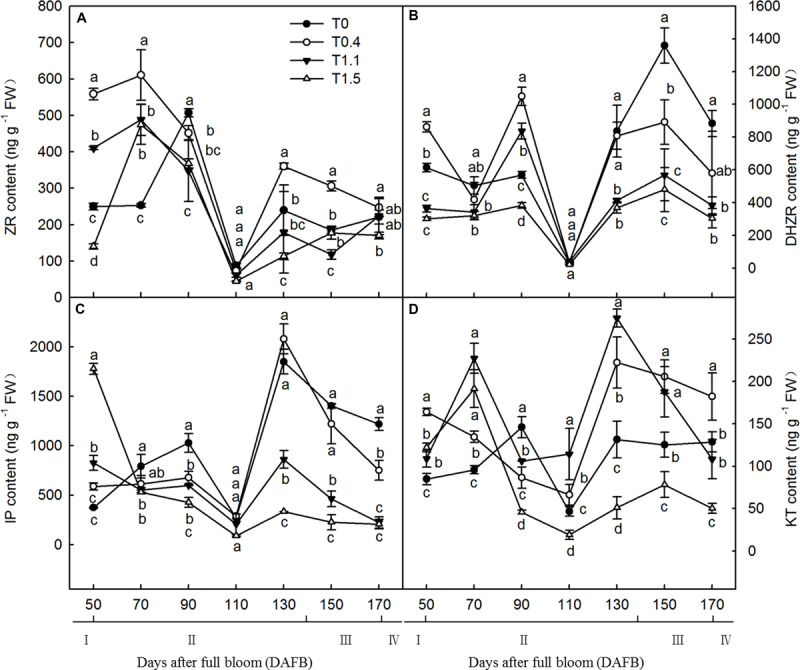
Changes in the ZR **(A)**, DHZR **(B)**, IP **(C)**, and KT **(D)** content of “Red Fuji” apple roots under different crop load treatments from 50 to 170 DAFB. Data are means ± SD of three replicate samples. Treatments: T0, the crop load levels of 0 fruits cm^–2^ TCSA; T0.4, the crop load levels of 0.4 fruits cm^–2^ TCSA; T1.1, the crop load levels of 1.1 fruits cm^–2^ TCSA; T1.5, the crop load levels of 1.5 fruits cm^–2^ TCSA. I, fruit set stage; II, fruit growth stage; III, fruit ripening stage; IV, fruit harvest stage. Different letters indicate significant differences between treatments, according to one-way ANOVA followed by Tukey’s multiple range test at *P*_0.05_ level.

Crop load significantly affected endogenous hormonal levels in roots, and root CTK content exhibited a bimodal curve throughout the fruit development stages (Figure 4). At both 90 and 130 DAFB, the ZR contents were significantly higher in lower and non-crop load treatments than in higher crop load treatments. The first peak appeared from 70 to 90 DAFB, and the second peak appeared from 110 to 130 DAFB; those of the four treatments appeared at different periods (Figure 4A). The root IP content also exhibited a bimodal curve throughout the fruit development period, with two peaks appearing at 90 and 130 DAFB. At both 90 and 130 DAFB, the root IP contents were significantly higher in T0 and T0.4 than in T1.1 and T1.5. Lower crop load treatments showed higher IP content, which decreased with increasing crop load (Figure 4B). The DHZR contents in all treatments increased after 70 DAFB, with the first peak at 90 DAFB and the second peak at 150 DAFB. The DHZR content during the first peak was significantly higher in T0.4 than the other treatments, whereas T1.5 had the lowest level. The DHZR content decreased with increasing crop load, and T0 exhibited the highest level among the treatments during the second peak (Figure 4C). Root KT content also exhibited a bimodal distribution, except for T0.4. The first peak appeared from 70 to 90 DAFB, and the second peak appeared from 130 to 150 DAFB. The KT contents in T1.1 and T1.5 at the first peak were significantly higher than those in the other treatments. However, after 90 DAFB, the KT content was significantly lower in T1.5 than in the other treatments (Figure 4D).

### Effects of Different Crop Loads on Root Auxin Content

**FIGURE 5:**
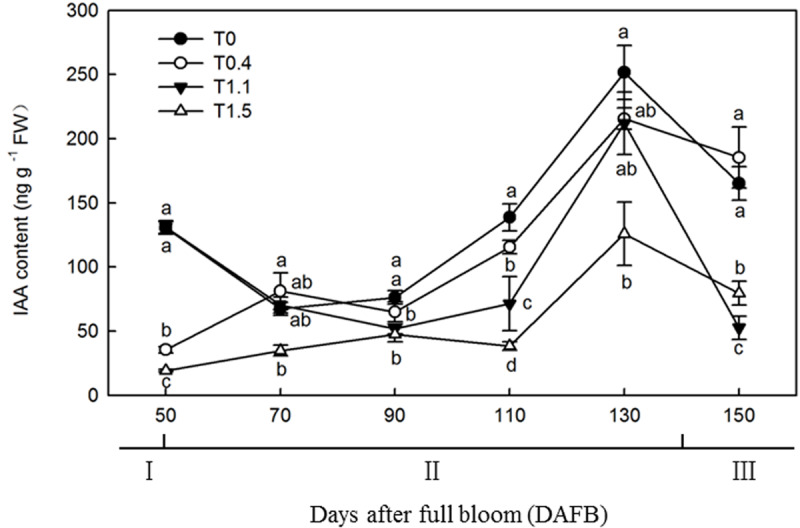
Changes in the IAA content of “Red Fuji” apple roots under different crop load treatments from 50 to 150 DAFB. Data are means ± SD of three replicate samples. Treatments: T0, the crop load levels of 0 fruits cm^–2^ TCSA; T0.4, the crop load levels of 0.4 fruits cm^–2^ TCSA; T1.1, the crop load levels of 1.1 fruits cm^–2^ TCSA; T1.5, the crop load levels of 1.5 fruits cm^–2^ TCSA. I, fruit set stage; II, fruit growth stage; III, fruit ripening stage. Different letters indicate significant differences between treatments, according to one-way ANOVA followed by Tukey’s multiple range test at *P*_0.05_ level.

The changes in IAA content were similar among the four crop loads, with unimodal curves peaking at 130 DAFB (Figure 5). At 130 DAFB, the IAA content was significantly higher in T0 than T1.1 and T1.5. No significant differences were observed in IAA contents between T0.4 and T0 or T1.1, but the IAA contents were significantly higher than that in T1.5 treatment (*p* < 0.05). The IAA content in T1.5 was significantly lower than that in the other treatments throughout the observation period (*p* < 0.05). At 110 DAFB, the IAA contents differed significantly among the four treatments. The IAA content was significantly higher in T0 than in the other treatments, and was significantly higher in T0.4 than in T1.1 and T1.5, whereas the IAA content was significantly lower in T1.5 than in the other treatments (*p* < 0.05).

### Effects of Different Crop Loads on Root GA_3_ Content

**FIGURE 6:**
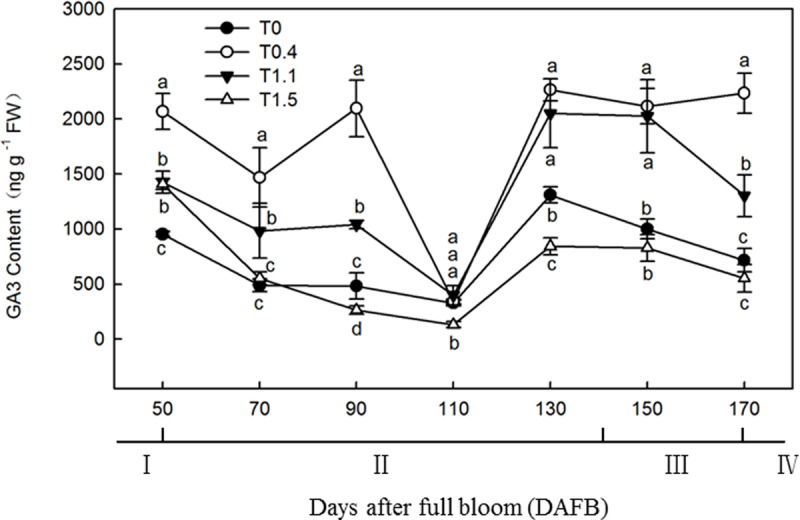
Changes in the GA3 content of “Red Fuji” apple roots under different crop load treatments from 50 to 170 DAFB. Data are means ± SD of three replicate samples. Treatments: T0, the crop load levels of 0 fruits cm^–2^ TCSA; T0.4, the crop load levels of 0.4 fruits cm^–2^ TCSA; T1.1, the crop load levels of 1.1 fruits cm^–2^ TCSA; T1.5, the crop load levels of 1.5 fruits cm^–2^ TCSA. I, fruit set stage; II, fruit growth stage; III, fruit ripening stage; IV, fruit harvest stage. Different letters indicate significant differences between treatments, according to one-way ANOVA followed by Tukey’s multiple range test at *P*_0.05_ level.

The root GA_3_ content exhibited a bimodal distribution through the fruit development stage in T0.4 and T1.1, and a unimodal distribution in T0 and T1.5 (Figure 6). In T0.4 and T1.1, the first GA_3_ content peak occurred at 90 DAFB; the GA_3_ content was significantly higher in T0.4 than the other treatments, and was significantly higher in T1.1 than in T0 and T1.5 (*p* < 0.05). The GA_3_ content also peaked at 130 DAFB. By contrast, the lowest GA_3_ contents were observed at 110 DAFB for all four treatments. At 130 DAFB, the GA_3_ content was significantly higher in T0.4 and T1.1 than in T0 and T1.5, and was significantly higher in T0 than in T1.5 (*p* < 0.05).

## Discussion

We examined the effects of crop load on growth and phytohormone contents in Fuji/SH40/Baleng Crabapple roots. Pome fruit trees exhibit irregular root growth patterns, with periods of active growth alternating with less active growth periods (Reig et al., 2013). In the present study, root growth dynamics exhibited bimodal curves, with decreases at 110 DAFB and a strong increase at 130 DAFB. Previous studies have shown that the fruit and vegetative growth of trees are affected by crop load, and that both shoot length and root growth are significantly restricted by excessive crop load (Pallas et al., 2018). These changes are induced by alterations in fixed carbon allocation to roots, because fruits are a major carbohydrate sink. Thus, during the most active period of fruit development, root growth parameters vary inversely with crop load. For example, Abrisqueta et al. (2017) reported that root growth activity was higher in non-fruiting trees than in fruiting trees. Similarly, decreases in root development were related to crop load in the present study; root growths was significantly higher in trees with low crop load than in those with high crop load. Thus, root growth was inhibited by crop load, possibly due to the absence of competition with fruit growth.

Cytokinins inhibit the initiation of root primordia but have a positive effect on root elongation, and IAA plays an important role in root initiation and elongation (Debi et al., 2005). GA has been shown to control root growth at a considerably lower concentration than is necessary for controlling shoot growth (Tanimoto, 1994). In the present study, increases in root growth corresponded with increases in hormones contents during the fruit development stage. Root development dynamics appear to have been mediated by hormones under different crop loads, such that the source–sink ratio and carbohydrate allocation affected hormone signaling during root development. High crop loads have been reported to decrease aerial part IAA content and basipetal transportation to roots, affecting root initiation and development (Casimiro et al., 2003; Van Hooijdonk et al., 2010). Our findings demonstrate that excessive crop load reduces root IAA content, thereby causing a decline in root growth. By extension, IAA content may also be positively correlated with root growth.

Reductions in hormone contents inhibits the development of root primordia, weakens growth vigor, and results in less root growth (Aloni et al., 2010). In the present study, the CTK and GA_3_ levels in roots of the high crop load treatments were also the lowest among all treatments. CTKs and GA_3_ in roots may be regulated by IAA content and transport, considering that less vegetative growth affects the IAA level in both aerial and belowground parts of apple trees, resulting in less vigorous growth (Van Hooijdonk et al., 2010). Considering the relatively high levels of CTKs and GA_3_ and the low quantity of roots in non-fruiting trees, an imbalance between hormone content and growth may be induced by undetected root growth in minirhizotrons. It can be assumed that the active growth in aerial parts provided abundant IAA to the roots, stimulating CTK and GA_3_ synthesis. Studies have shown that exogenous CTKs enhance root elongation but have side effects on the initiation of root primordia, which differed from our study (Mao et al., 2018). However, unlike IP and DHZR, ZR, and KT did not correlate well with the root growth under different crop loads at 130 DAFB, but the highest crop load treatment did have the lowest root growth rate. It was reported that ZR and KT showed significant inhibitory effects on adventitious root formation (Kuroha et al., 2002). The inhibition of adventitious root formation by CTKs occurs during the induction phase of root cell division. During the induction phase, IAA induces adventitious root formation in apple cuttings and seems to interact antagonistically with CTKs to control the initiation of adventitious roots (De Klerk et al., 1995). Endogenous CTKs along with IAA and GA_3_ may work together to affect root development, masking the inhibitory effect of CTKs (Hayat et al., 2019). The regulation effect of phytohormones on root development is complex, and the functions of endogenous hormones are still need to be further studied.

Tworkoski and Miller (2007) reported that GA_3_ negatively regulated radial growth of roots; thus, the higher concentration of GA_3_ may have decreased radial root growth, resulting in a long and thin root phenotype. In this study, the low- or non-fruiting treatments resulted in a higher GA_3_ content and higher root length density, which increased strongly after 130 DAFB; by contrast, root surface area and volume density did not increase as intensely as root length density. These differences may have been induced by the high root GA_3_ content. We conclude that the differences in phytohormone levels could be responsible for differences in root growth vigor under different crop loads. In the present study, IAA, CTK, and GA_3_ contents were positively correlated with root growth vigor in the different crop load treatments. The decline in root growth at 110 DAFB occurred when IAA, CTKs, and GA_3_ reached their lowest levels, whereas hormone contents increased rapidly at 130 DAFB, and were subsequently maintained at relatively high levels. Root growth decreased after the root growth peak in fall along with the increase in crop load, which was positively correlated with hormone contents.

## Conclusion

In conclusion, the results of this study confirm that fruit plays an important role in restricting root growth and hormone contents in apple. Trees with a low crop load showed more active root growth than trees with high crop loads. Excessive crop loads limited root growth and IAA, CTK, and GA_3_ contents during the fruit growth phase. Our data also provided evidence that root growth dynamics and IAA, IP, DHZR, ZR, and GA_3_ contents were positively correlated. We suggest that the decrease in IAA, CTK, and GA_3_ levels in roots could be considered compliant with the root growth restriction caused by crop load.

## Data Availability Statement

The datasets generated for this study are available on request to the corresponding author.

## Author Contributions

BL, YS, and JX conceived and designed the experiments. YS performed the experiments with assistance from ZL, XZ, BY, and SZ. BL performed the data analyses and wrote the manuscript. BL and JX provided financial support and helped to perform the analysis with constructive discussions.

## Conflict of Interest

The authors declare that the research was conducted in the absence of any commercial or financial relationships that could be construed as a potential conflict of interest.
